# Development of the OncoScore: A Diagnostic Prediction Score for Previously Unrecognized Malignancy in Patients with Acute Pulmonary Embolism

**DOI:** 10.3390/cancers18213440

**Published:** 2026-10-24

**Authors:** Aleksandra Kozic, Sonja Salinger, Boris Dzudovic, Jovan Matijasevic, Marija Benic, Vladimir Miloradovic, Ema Jevtic, Tamara Kovacevic Preradovic, Ljiljana Kos, Bjanka Bozovic, Irena Mitevska, Marijan Bosevski, Ana Kovacevic-Kuzmanovic, Aleksandar Neskovic, Bojan Mitrovic, Srdjan Kafedzic, Slobodan Obradovic

**Affiliations:** 1 Medical Faculty of the Military Medical Academy, University of Defence, 17 Crnotravska, 11000 Belgrade, Serbiasloba.d.obradovic@gmail.com (S.O.); 2 Clinic of Cardiology, University Clinical Center Nis, 18000 Nis, Serbia; 3 Faculty of Medicine, University of Nis, 18000 Nis, Serbia; 4 Clinic for Emergency Internal Medicine, Military Medical Academy, 11000 Belgrade, Serbia; 5 Clinic for Intensive Care Medicine and Pulmonary Vascular Diseases, Institute for Pulmonary Diseases Vojvodina, 21204 Sremska Kamenica, Serbia; 6 Faculty of Medicine, University of Novi Sad, 21000 Novi Sad, Serbia; 7 Clinic of Cardiology, University Clinical Center Kragujevac, 34000 Kragujevac, Serbia; 8 Faculty of Medical Sciences, University of Kragujevac, 34000 Kragujevac, Serbia; 9 Clinic of Cardiology, University Clinical Center of the Republic of Srpska, 78000 Banja Luka, Republic of Srpska, Bosnia and Herzegovina; 10 Faculty of Medicine, University of Banja Luka, 78000 Banja Luka, Republic of Srpska, Bosnia and Herzegovina; 11 Clinic of Cardiology, Clinical Center of Montenegro, 81000 Podgorica, Montenegro; 12 University Clinic for Cardiology and Cardiovascular Surgery, National Center for Cardiovascular Diseases, 1000 Skopje, North Macedonia; 13 Faculty of Medicine, Ss. Cyril and Methodius University in Skopje, 1000 Skopje, North Macedonia; 14 Department for Internal Medicine, General Hospital Pancevo, 26000 Pancevo, Serbia; 15 Department of Cardiology, Clinical Hospital Center Zemun, 11080 Belgrade, Serbia; 16 Faculty of Medicine, University of Belgrade, 11000 Belgrade, Serbia; 17 Clinic for Cardiology, Military Medical Academy, 11000 Belgrade, Serbia

**Keywords:** pulmonary embolism, malignancy, cancer screening, diagnostic score, OncoScore

## Abstract

**Background/Objectives:** Pulmonary embolism (PE) may represent the first clinical manifestation of a previously unrecognized malignancy. We aimed to develop the OncoScore, a diagnostic prediction score for estimating the probability of previously unrecognized malignancy being diagnosed during the index hospitalization for acute PE. **Methods:** We conducted a retrospective diagnostic prediction model derivation study using prospectively collected data from the multicenter Regional Pulmonary Embolism Registry (REPER). Candidate predictors were assessed using logistic regression and Chi-square Automatic Interaction Detection (CHAID) analysis, and independent predictors were incorporated into the OncoScore. Discrimination was assessed by receiver operating characteristic (ROC) analysis, calibration by calibration slope and a calibration plot, and internal validation by bootstrap resampling. Diagnostic performance measures were calculated using the cut-off value determined by the Youden index. **Results:** Among 2615 patients with acute PE, 91 had newly diagnosed malignancy, 265 had previously known malignancy, and 2259 had no malignancy. The final OncoScore included hemoglobin, C-reactive protein, platelet count, and leukocyte count, with a total score ranging from 0 to 7 points. The OncoScore yielded an area under the curve (AUC) of 0.702 (95% confidence interval [CI] 0.653–0.751; *p* < 0.001). Bootstrap internal validation of the fixed score yielded an optimism-corrected AUC of 0.7013 and an optimism-corrected calibration slope of 1.015. When predictor selection and CHAID cut-point determination were repeated within the bootstrap procedure, the optimism-corrected AUC was 0.6560. Patients were stratified as low (≤2), intermediate (3–4), and high risk (≥5), with observed malignancy prevalences of 1.1%, 5.5%, and 10.8%, respectively. At a cut-off of ≥3 points, sensitivity was 87.5%, specificity 46.5%, positive predictive value 6.4%, and negative predictive value 98.9%. **Conclusions:** The OncoScore is a simple laboratory-based prediction tool that showed moderate discrimination and identified clinically distinct risk strata for malignancy diagnosed during the index hospitalization for acute PE. The score should be considered a preliminary derivation-stage model, and independent external validation is required before clinical implementation.

## Simple Summary

Acute pulmonary embolism may be the first clinical manifestation of a previously unrecognized malignancy. However, identifying which patients may require further investigation for malignancy remains challenging. In this multicenter study, we developed the OncoScore, a simple diagnostic score based on routinely available laboratory parameters measured at hospital admission, to estimate the likelihood of previously unrecognized malignancy in patients with acute pulmonary embolism. The score incorporates hemoglobin, C-reactive protein, platelet count, and leukocyte count and stratifies patients into low-, intermediate-, and high-risk groups. The OncoScore showed moderate discrimination and identified clinically distinct risk groups with progressively increasing observed prevalence of newly diagnosed malignancy. As a simple bedside tool, it may help clinicians stratify suspicion for previously unrecognized malignancy and support a more individualized approach to additional diagnostic evaluation during the index hospitalization. External validation is required before the score can be used to guide clinical decision-making.

## 1. Introduction

Pulmonary embolism (PE) is a potentially life-threatening condition with a well-established association with malignancy [1]. Cancer-associated thrombosis represents a complex clinical entity arising from the interplay between prothrombotic tumor biology, anticancer therapies, systemic inflammation, and patient-related factors, resulting in both an increased incidence and a worse prognosis of PE in this population [1,2,3,4]. Patients with cancer-associated PE experience higher mortality rates and worse clinical outcomes compared with patients without malignancy [5,6]. Furthermore, PE may precede the diagnosis of malignancy and, in some patients, represent the first clinically apparent manifestation of cancer [7,8]. Previous studies have shown that approximately 2–10% of patients presenting with venous thromboembolism (VTE) are subsequently diagnosed with a previously unrecognized malignancy, with the highest risk observed during the first year after the thromboembolic event [8,9].

Despite the well-established association between PE and malignancy, there is no universally accepted strategy [10,11,12,13] for systematic cancer screening in patients presenting with acute PE. Current recommendations are mainly derived from studies of unprovoked VTE, and most guidelines support a limited initial screening approach, as extensive screening strategies, including computed tomography (CT)- or positron emission tomography/computed tomography (PET/CT)-based approaches, have not been shown to provide a clear survival benefit [10,11,12,13]. Therefore, recent reviews emphasize the need for a more individualized, risk-based approach in order to identify patients most likely to benefit from additional diagnostic evaluation while avoiding unnecessary costs, radiation exposure, invasive procedures, and psychological burden [11,14].

In this context, a simple and readily applicable tool for estimating the probability of malignancy diagnosed during the hospitalization for acute PE could be of substantial clinical value. Existing prediction models have primarily been developed to identify occult cancers diagnosed during follow-up after VTE [14,15,16]. In contrast, the present study addresses a distinct clinical question: the identification of malignancy diagnosed during the index hospitalization for acute pulmonary embolism. These approaches should be regarded as complementary, as assessment during the index hospitalization does not capture malignancies diagnosed during subsequent follow-up.

The aim of the present study was to identify predictors of previously unrecognized malignancy in patients presenting with acute PE and to integrate them into a diagnostic prediction score. The intended purpose of the score is to estimate the likelihood of malignancy being diagnosed during the index hospitalization for acute PE and to stratify patients according to their observed risk.

## 2. Materials and Methods

### 2.1. Study Design and Population

This was a retrospective diagnostic prediction model derivation study, based on prospectively collected registry data of patients hospitalized with acute PE. In the Regional Pulmonary Embolism Registry (REPER), a multicenter prospective registry consisting of eight hospitals (seven university hospitals and one general hospital), consecutive PE patients were enrolled from January 2015 to December 2025. No prospective sample-size calculation was performed; all eligible patients available during the study period were included. Acute PE was diagnosed according to the European Society of Cardiology (ESC) algorithm, with computed tomography pulmonary angiography (CTPA) performed in all patients [17].

For the purpose of the present analysis, patients were categorized into three predefined groups according to the presence and timing of malignant disease diagnosis:

1. Patients with newly diagnosed malignancy during hospitalization for acute PE. Malignancy was diagnosed based on imaging findings highly suggestive of cancer, followed by invasive diagnostic procedures and histopathological confirmation whenever patient’s clinical condition allowed.
2. Patients with previously known active malignancy. Active malignancy was defined as histologically confirmed cancer that was previously known and treated or monitored within the preceding six months.
3. Patients with PE and without malignancy diagnosed during the index hospitalization. In this group, patients underwent a routine initial evaluation including detailed medical history, routine laboratory testing, and thoracic CT with CTPA at admission. In the majority of patients older than 50 years without an identifiable provoking factor for PE, abdominal and/or gynecological ultrasound was also performed as part of the initial evaluation. Based on these findings, the decision to pursue additional investigations for suspected malignancy was made individually according to the treating physician’s clinical judgment and local clinical practice.

For score derivation, patients with newly diagnosed malignancy during the index hospitalization for acute PE constituted the derivation group, whereas patients without malignancy diagnosed during the index hospitalization served as the reference group. Patients with previously known active malignancy were not included in score derivation and were analyzed separately in an exploratory analysis.

### 2.2. Candidate Predictors

Based on clinical relevance and the previous literature [15,18], candidate variables included demographic characteristics (age and sex), comorbidities, body mass index (BMI), D-dimer, renal function parameters, and routinely available hematological and inflammatory laboratory markers. Laboratory predictors were based on routinely available baseline blood samples obtained at hospital admission for acute PE, before subsequent diagnostic evaluation for malignancy.

### 2.3. Statistical Analysis

Categorical variables were presented as frequencies and percentages, while continuous variables were expressed as means with standard deviations or medians with first and third quartiles (Q1–Q3), depending on data distribution. Differences between groups were assessed using the chi-square test for categorical variables and one-way analysis of variance (ANOVA) or the Kruskal–Wallis test for continuous variables, as appropriate. Univariate logistic regression analysis of candidate variables was initially performed to identify variables associated with newly diagnosed malignancy. Variables significantly associated in the univariate analysis were subsequently analyzed using Chi-square Automatic Interaction Detection (CHAID) classification tree analysis to determine optimal cut-off values. CHAID analyses used automatic maximum tree depth, minimum parent and child node sizes of 100 and 50 cases, respectively, splitting and merging significance levels of 0.05, Pearson chi-square testing with Bonferroni adjustment, and initial division of continuous predictors into 10 intervals. Integer point values were assigned to the resulting CHAID-defined categories according to the observed prevalence of newly diagnosed malignancy, with higher point values assigned to categories with higher malignancy prevalence. The resulting point-coded OncoScore component categories were subsequently evaluated using univariate and multivariable logistic regression analyses to assess their association with newly diagnosed malignancy and their independent contributions to the score. Logistic regression results were expressed as odds ratios (ORs) with 95% confidence intervals (CIs). The total OncoScore was calculated as the sum of the individual component points. Receiver operating characteristic (ROC) curve analysis was used to assess the discriminative performance of the derived score, quantified by the area under the curve (AUC). The optimal cut-off value for the OncoScore was determined using the Youden index. Sensitivity, specificity, positive predictive value (PPV), and negative predictive value (NPV) were reported with 95% exact binomial CIs. Overall predictive accuracy was assessed using the Brier score. Internal validation was performed using 2000 bootstrap resamples. For the final fixed OncoScore, optimism-corrected estimates of the AUC, Brier score, calibration slope, and calibration-in-the-large were calculated. Calibration was additionally assessed graphically by comparing predicted and observed malignancy risks across individual OncoScore values, with 95% exact binomial CIs for observed event proportions. To assess optimism introduced during model development, an additional bootstrap procedure repeated the original univariable predictor-selection step and CHAID cut-point determination within each resample, followed by refitting of the resulting categorized logistic model. Selection frequencies and cut-point stability were examined, and optimism-corrected discrimination and overall predictive accuracy were estimated for this repeated development procedure. Clinical utility was explored using decision-curve analysis by comparing the net benefit of the OncoScore with strategies of investigating all patients or investigating none across a range of threshold probabilities. As a post hoc sensitivity analysis, the incremental predictive value of BMI was evaluated by comparing the OncoScore alone with models additionally including continuous or categorical BMI. Differences in discrimination were assessed by paired comparison of AUCs, while model fit and overall predictive accuracy were evaluated using likelihood-ratio testing and the Brier score, respectively. A two-sided *p*-value < 0.05 was considered statistically significant. Primary statistical analyses were performed using IBM SPSS Statistics version 31 (IBM Corp., Armonk, NY, USA), while additional internal-validation, calibration, and sensitivity analyses were performed in R version 4.6.1 (R Foundation for Statistical Computing, Vienna, Austria).

Patients with missing values for one or more OncoScore components were excluded from analyses requiring calculation of the total OncoScore (complete-case analysis).

## 3. Results

### 3.1. Study Population

A total of 2615 patients with acute pulmonary embolism were included in the present analysis. According to the presence and timing of malignant disease, 91 (3.5%) patients had malignancy newly diagnosed during hospitalization for pulmonary embolism, 265 (10.1%) patients had previously known active malignancy, and 2259 (86.4%) patients had no evidence of malignant disease during the index hospitalization.

### 3.2. Baseline Characteristics

Baseline demographic, clinical, and laboratory characteristics of the study population according to malignancy status are presented in Table 1.

Age and sex distributions were similar across the three groups. Among the evaluated comorbidities, arterial hypertension was more common in patients without malignancy than in those with newly diagnosed or previously known malignancy (*p* = 0.021), whereas chronic heart failure, chronic lung disease, coronary artery disease, and diabetes mellitus were similarly distributed across the groups. Renal impairment, defined as estimated glomerular filtration rate (eGFR) < 60 mL/min/1.73 m^2^, was similarly distributed across the groups, whereas severe renal impairment (eGFR < 30 mL/min/1.73 m^2^) was more frequent among patients with previously known malignancy (*p* = 0.002).

BMI categories differed significantly between groups (*p* < 0.001), with overweight and obesity being more common in patients without malignancy.

The distribution of patients across ESC mortality risk categories was significantly different (*p* < 0.001), with cancer-associated PE more frequently represented in higher-risk strata.

Anemia was substantially more frequent in both cancer groups, particularly in patients with previously known malignancy (*p* < 0.001). Similarly, D-dimer and C-reactive protein (CRP) concentrations differed significantly among the three groups (*p* = 0.048 and *p* < 0.001, respectively), with higher values observed in both malignancy groups than in patients without malignancy. In contrast, leukocyte and platelet counts did not differ significantly in the descriptive analysis.

No statistically significant differences were observed between patients with newly diagnosed and previously known malignancy in the analyzed baseline characteristics, with the exception of hemoglobin concentration on admission, which was significantly lower in patients with previously known malignancy (*p* = 0.015).

### 3.3. Univariate Logistic Regression Analysis

Univariate logistic regression analysis identified four continuous laboratory variables (CRP, leukocyte count, platelet count, and hemoglobin) and BMI as significant predictors of newly diagnosed malignancy, whereas age, sex, renal function, D-dimer, and the evaluated comorbidities were not significantly associated with the outcome (Table 2).

### 3.4. Derivation of the OncoScore

Based on the results of the univariate logistic regression analysis, four continuous laboratory variables (CRP concentration, leukocyte count, platelet count, and hemoglobin concentration) were selected for CHAID classification tree analysis to determine the optimal cut-off values, which were subsequently used to categorize laboratory parameters for score development. Although identified as significant predictor of newly diagnosed malignancy, BMI was not included in score development because of the substantial proportion of missing values. The resulting CHAID-derived categories of the OncoScore components were as follows:

- CRP concentration: ≤56 mg/L = 0 points; >56 mg/L = 1 point.
- Platelet count: ≤225 × 10^9^/L = 0 points; >225 × 10^9^/L = 1 point.
- Leukocyte count: ≤16 × 10^9^/L = 0 points; >16 × 10^9^/L = 2 points.
- Hemoglobin concentration: ≤103 g/L = 3 points; >103–≤142 g/L = 2 points; >142 g/L = 0 points.

The total OncoScore ranged from 0 to 7 points. Univariate logistic regression analysis demonstrated that all four OncoScore components were significantly associated with newly diagnosed malignancy (Table 3).

In multivariable logistic regression analysis adjusted for age and sex, all four OncoScore components remained independently associated with newly diagnosed malignancy (Table 4). The hemoglobin component demonstrated the strongest independent association with progressively higher odds of malignancy for concentrations >103–≤142 g/L (OR 3.82, 95% CI 1.78–8.18, *p* < 0.001) and ≤103 g/L (OR 7.41, 95% CI 3.11–17.64, *p* < 0.001), compared with >142 g/L. CRP, platelet count, and leukocyte count components also remained significant independent predictors.

### 3.5. Risk Categories of the OncoScore

According to score distribution and observed malignancy prevalence, three descriptive risk strata were defined:

- Low risk: OncoScore ≤ 2;
- Intermediate risk: OncoScore 3–4;
- High risk: OncoScore ≥ 5.

The observed prevalence of newly diagnosed malignancy increased stepwise across the three risk categories, together with a corresponding increase in mean model-predicted probability (Table 5).

The OncoScore was calculable in 2189 of 2350 patients (93.1%) with complete data for all four score components; 161 patients were excluded because of missing values for one or more score components (3 with newly diagnosed malignancy and 158 without malignancy). Missing values were present for CRP in 106 patients (4.5%), platelet count in 69 (2.9%), hemoglobin in 20 (0.9%), and leukocyte count in 20 (0.9%); individual categories were not mutually exclusive because some patients had more than one missing component. The proportion of patients with incomplete OncoScore data did not differ significantly between those with newly diagnosed malignancy and those without malignancy (3.3% vs. 7.0%, *p* = 0.171). Patients with incomplete data were slightly younger than complete-case patients (61.8 ± 16.3 vs. 64.6 ± 16.0 years, *p* = 0.032), whereas sex distribution was similar (male sex 46.0% vs. 47.4%, *p* = 0.729). Missingness for all candidate predictors is reported in Supplementary Table S1; missing data were generally limited, except for BMI category (28.3%) and D-dimer (7.6%).

### 3.6. Discriminatory Performance, Internal Validation, and Calibration of the OncoScore

**Figure 1:**
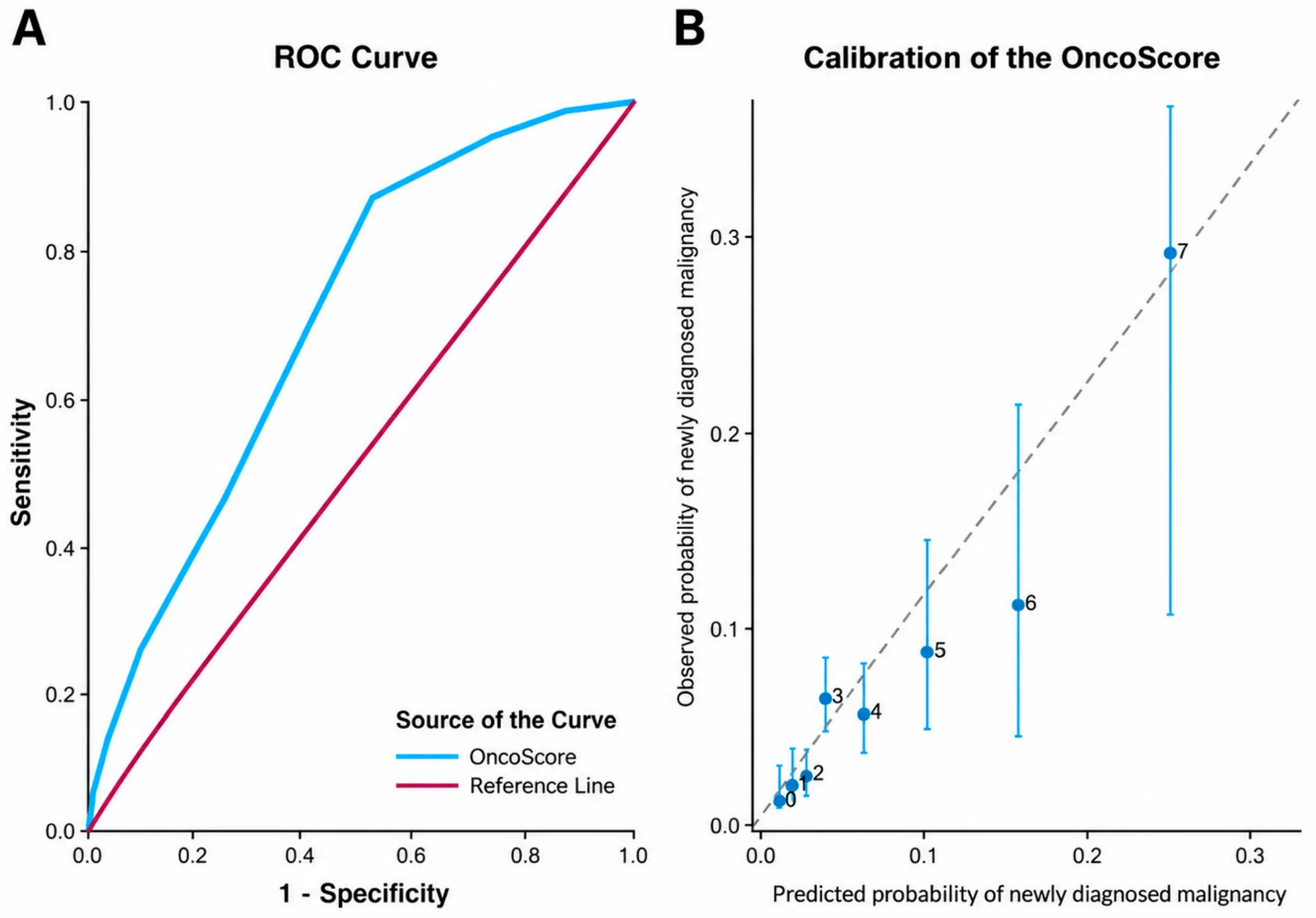
(**A**) Receiver operating characteristic (ROC) curve of the OncoScore for prediction of newly diagnosed malignancy. (**B**) Calibration plot showing observed versus predicted probabilities of newly diagnosed malignancy across individual OncoScore values, with 95% confidence intervals. Numbers indicate individual OncoScore values. The diagonal line represents perfect calibration.

ROC curve analysis was performed in the 2189 patients with complete OncoScore data to evaluate the ability of the OncoScore to identify newly diagnosed malignant disease. The AUC was 0.702 (95% CI 0.653–0.751; *p* < 0.001) (Figure 1A). The final model achieved a Brier score of 0.038.

Bootstrap internal validation of the fixed final OncoScore using 2000 resamples yielded an optimism-corrected AUC of 0.7013 and an optimism-corrected Brier score of 0.03776. The optimism-corrected calibration slope was 1.015, and calibration-in-the-large was 0.006. The calibration plot across individual OncoScore values is shown in Figure 1B. When predictor selection and CHAID cut-point determination were repeated within the bootstrap procedure, the categorized model had an apparent AUC of 0.7051, with mean optimism of 0.0491 and an optimism-corrected AUC of 0.6560; the optimism-corrected Brier score was 0.03840.

Decision-curve analysis showed a higher net benefit for the OncoScore than strategies of investigating all patients or investigating none across a range of low threshold probabilities (Supplementary Figure S1).

In post hoc sensitivity analyses, addition of categorical BMI, available in 71.7% of patients, increased the AUC from 0.7196 to 0.7372 (ΔAUC = 0.0176; paired DeLong *p* = 0.2883). Among patients with available continuous BMI data (57.9% of the derivation cohort), addition of BMI increased the AUC from 0.7118 to 0.7486 (ΔAUC = 0.0368; paired DeLong *p* = 0.0576).

To further evaluate the clinical applicability of the OncoScore, the optimal cut-off value was determined using the Youden index. Patients with an OncoScore ≥ 3 (corresponding to the intermediate- and high-risk categories) were considered test-positive. Sensitivity, specificity, PPV, and NPV for the identification of newly diagnosed malignancy are presented in Table 6.

### 3.7. Exploratory Analysis in Patients with Previously Known Malignancy

The performance of the OncoScore was further evaluated in patients with previously known malignancy. In an exploratory analysis comparing this group of patients with patients without malignancy, the OncoScore yielded an AUC of 0.660 (95% CI 0.627–0.694; *p* < 0.001) (Supplementary Figure S2).

To assess the potential impact of recent surgery on score performance in the group of patients with previously known malignancy, a sensitivity analysis was performed after excluding patients who had undergone any surgical procedures within six months prior to pulmonary embolism. The discriminative ability of the score was slightly improved, with an AUC of 0.672 (95% CI 0.632–0.713; *p* < 0.001) (Supplementary Figure S3).

### 3.8. Performance of the OncoScore According to Detectability on Thoracic CTPA

The anatomical distribution of the 91 newly diagnosed malignancies is presented in Table 7. For subgroup analysis, malignancies were additionally classified according to whether the primary tumor or malignant lesions suggestive of an underlying malignancy could be detected on thoracic CTPA performed as part of the diagnostic work-up for acute PE. Overall, 45 of 91 malignancies were classified as CTPA-detectable, whereas 46 were not detectable on thoracic CTPA.

**Figure 2:**
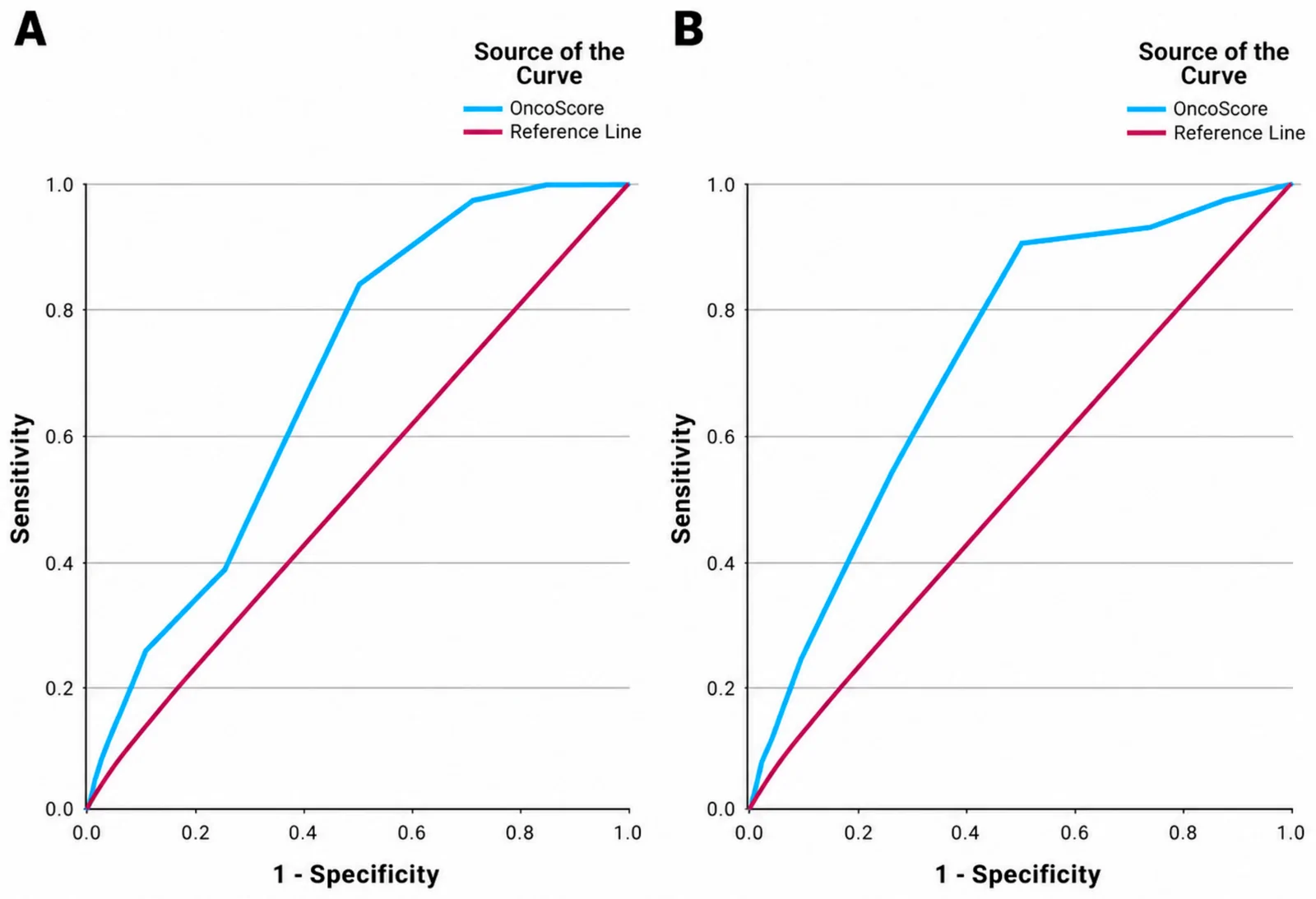
(**A**) Receiver operating characteristic (ROC) curve of the OncoScore for prediction of newly diagnosed CTPA-detectable malignancies in patients with pulmonary embolism. (**B**) ROC curve of the OncoScore for prediction of newly diagnosed non-CTPA-detectable malignancies in patients with pulmonary embolism.

In an exploratory subgroup analysis, separate ROC analyses were performed for CTPA-detectable and non-CTPA-detectable newly diagnosed malignancies, each compared with patients without malignancy. In patients with newly diagnosed CTPA-detectable malignancies, the AUC was 0.681 (95% CI 0.614–0.749; *p* < 0.001) (Figure 2A).

In patients with newly diagnosed non-CTPA-detectable malignancies, the discriminatory performance was slightly higher, with an AUC of 0.723 (95% CI 0.654–0.792; *p* < 0.001) (Figure 2B).

## 4. Discussion

### 4.1. Main Findings and Clinical Implications

In the present study, we developed the OncoScore, a simple laboratory-based prediction score for identifying previously unrecognized malignancy in patients presenting with acute pulmonary embolism. The score demonstrated moderate discriminatory performance (AUC 0.702), identified three distinct risk categories, and achieved a high NPV, suggesting potential utility for identifying patients with a lower observed prevalence of malignancy diagnosed during the index hospitalization.

However, when the entire model-development procedure, including predictor selection and CHAID cut-point determination, was repeated within bootstrap resampling, the optimism-corrected AUC decreased to 0.656, indicating instability in these data-driven development steps and supporting the interpretation of the OncoScore as a preliminary derivation-stage model.

The principal clinical value of the OncoScore lies in its potential to support decision-making regarding additional cancer evaluation during the index hospitalization for acute pulmonary embolism. Current recommendations for occult cancer evaluation after unprovoked VTE generally favor a limited screening approach, whereas specific guidance on how to stratify the need for additional oncological investigation during the index hospitalization for acute PE is lacking [10,11,13]. In our cohort, patients with an OncoScore ≤ 2 (low risk) had a prevalence of previously unrecognized malignancy of only 1.1%, while the score achieved a NPV of 98.9% using the ≥3-point cut-off. The ≥3-point cut-off was identified using the Youden index and therefore represents the statistically optimal threshold in the present derivation cohort. The three score strata should be interpreted as risk-stratification categories reflecting progressively higher observed malignancy prevalence. These findings suggest that the OncoScore may help identify patients in whom the diagnostic yield of extensive additional oncological evaluation during the index hospitalization is relatively low, although the clinical implications of these thresholds require further validation. The present study does not address the risk of malignancy diagnosed during subsequent follow-up. Importantly, a low OncoScore should not be interpreted as ruling out occult malignancy and should not, by itself, justify withholding otherwise clinically indicated cancer investigation or subsequent clinical follow-up. Conversely, the modest specificity and low PPV indicate that the OncoScore should not be used to confirm the presence of malignancy.

Importantly, the objective of the present study differs from that of most previous studies investigating occult cancer after VTE. Whereas previous screening studies and prediction models generally evaluated occult cancers defined by diagnosis during the initial diagnostic work-up and/or subsequent follow-up period after VTE, our study specifically focused on malignancy identified during the index hospitalization for acute pulmonary embolism [7,14,15,16]. Therefore, the OncoScore should be viewed primarily as a diagnostic prediction tool aimed at identifying malignancy already detectable during the index PE hospitalization, rather than predicting cancer diagnosis during subsequent follow-up.

The clinical value of earlier detection of occult malignancy after VTE remains uncertain. More extensive screening strategies, including CT- or PET/CT-based approaches, may increase cancer detection but have not demonstrated a clear improvement in overall survival compared with limited screening strategies. Accordingly, the present findings should not be interpreted as evidence that OncoScore-guided diagnostic evaluation would improve survival. Its intended role is limited to diagnostic risk stratification during the index hospitalization and to supporting a more individualized approach to further investigation, rather than serving as a validated cancer-screening strategy, while any effect on patient outcomes requires prospective evaluation.

Data specifically addressing the prevalence of previously unrecognized malignancy diagnosed during the index hospitalization for acute pulmonary embolism are limited. In a PE-specific cohort reported by Felix et al., 8.4% of patients were diagnosed with previously unknown cancer either during the acute PE admission or during the subsequent two-year follow-up; however, this broader outcome is not directly comparable with the present study, which was restricted to malignancy diagnosed during the index hospitalization [18]. Previous studies restricted to unprovoked VTE have reported approximately 3.5% occult cancer detection during the initial diagnostic evaluation, but these populations are likewise not directly comparable with our all-comer acute-PE cohort [19].

Apart from laboratory variables, several baseline differences were observed between study groups. Among the baseline characteristics, arterial hypertension and overweight/obesity were more prevalent in patients without malignancy. Lower BMI in patients with malignancy may partly reflect cancer-associated weight loss and cachexia [20], while reduction in body weight may also be accompanied by lower arterial blood pressure [21]. These observations should, however, be regarded as descriptive and not as evidence of a causal relationship. Although preceding weight loss may be more clinically informative than a single BMI measurement in this context, data on weight loss before hospitalization were not systematically collected in the REPER and therefore could not be evaluated in the present analysis. Differences in severe renal impairment (eGFR < 30 mL/min/1.73 m^2^) were observed across the study groups, driven mainly by the higher prevalence among patients with previously known malignancy. This finding is consistent with previous observations in patients with cancer-associated pulmonary embolism and may partly reflect the effects of anticancer treatment on renal function [6,22]. Differences in ESC mortality-risk distribution were also observed and are consistent with previous reports demonstrating a more severe presentation and worse early outcomes of acute pulmonary embolism in patients with active malignancy [4,6].

### 4.2. Development and Biological Rationale of the OncoScore

**Figure 3:**
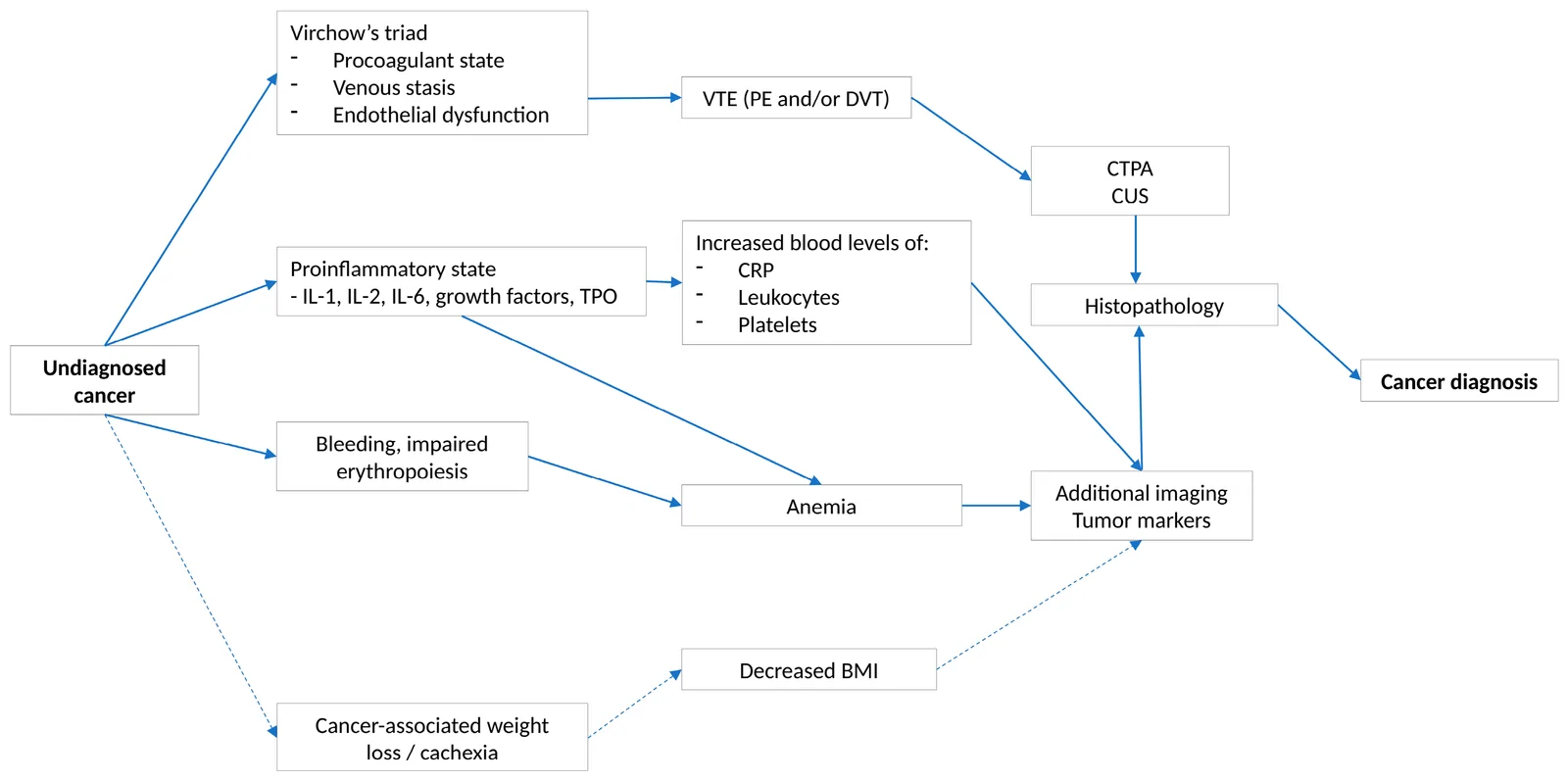
Proposed pathophysiological framework linking malignancy, cancer-associated inflammation and hypercoagulability, venous thromboembolism, and the laboratory and clinical features relevant to the OncoScore. Dashed arrows indicate variables biologically related to malignancy but not included in the final OncoScore. Abbreviations: BMI, body mass index; CRP, C-reactive protein; CTPA, computed tomography pulmonary angiography; CUS, compression ultrasonography; DVT, deep vein thrombosis; IL, interleukin; PE, pulmonary embolism; TPO, thrombopoietin; VTE, venous thromboembolism.

The OncoScore was developed using routinely available laboratory parameters available at hospital admission. Four variables remained independently associated with newly diagnosed malignancy and were incorporated into the final score: hemoglobin concentration, CRP concentration, platelet count, and leukocyte count. The biological plausibility of these findings is supported by the known pathophysiological relationship between malignancy, inflammation, and thrombosis [2]. Accordingly, routinely available laboratory parameters reflecting systemic inflammation, hematopoiesis, and coagulation activation may provide indirect evidence of malignancy-associated biological activity. Tumor cells promote activation of coagulation pathways through expression of procoagulant proteins, release of inflammatory mediators, and interactions with platelets, leukocytes, and endothelial cells. Consequently, systemic inflammatory activation and tumor-associated cytokine release may contribute to thrombocytosis, leukocytosis, elevated C-reactive protein concentrations, and anemia of chronic disease, all of which have previously been associated with malignant disease [23]. These interrelated pathways are summarized in Figure 3, which illustrates the proposed pathophysiological framework linking malignancy, cancer-associated inflammation and hypercoagulability, venous thromboembolism, and the laboratory abnormalities incorporated into the OncoScore. Notably, all four variables are routinely measured at hospital admission and require no additional testing, supporting the clinical applicability of the OncoScore in the acute clinical setting.

Hemoglobin showed the strongest independent association with malignancy diagnosed during the index hospitalization. This finding is consistent with the well-established pathophysiology of cancer-related anemia, which is multifactorial and may result from chronic inflammation, impaired erythropoiesis, altered iron metabolism, blood loss, nutritional deficiencies, and bone marrow involvement [24]. Chronic inflammation suppresses erythropoiesis through cytokine-mediated inhibition of erythropoietin production and iron utilization, while increased hepcidin expression further limits iron availability for red blood cell production [25]. Elevated CRP most likely reflects the systemic inflammatory response induced by tumor-derived cytokines and has been associated with active and more advanced malignant disease [26,27]. Thrombocytosis is largely mediated by IL-6-driven thrombopoietin production and has been linked to tumor progression and cancer-associated thrombosis [28,29]. Leukocytosis may likewise represent a manifestation of the systemic inflammatory response associated with malignancy [30]. In cancer populations, leukocytosis, particularly neutrophilia, has also been associated with an increased risk of venous thromboembolism [30]. However, these laboratory abnormalities are not specific to malignancy and may also be influenced by acute infection, recent surgery, bleeding, corticosteroid use, iron deficiency, chronic inflammatory conditions, renal or liver disease, and the acute stress response associated with PE.

Unlike several recent studies in patients with pulmonary embolism that identified elevated D-dimer concentrations as an independent predictor of subsequent cancer diagnosis, D-dimer was not significantly associated with previously unrecognized malignancy in our cohort [18,31]. This discrepancy may reflect differences in study populations, study endpoints, and model development strategies. While D-dimer primarily reflects coagulation activation and thrombus burden, the laboratory variables included in the OncoScore appear to capture broader manifestations of cancer-associated systemic inflammation and hematological disturbance.

Although BMI was significantly associated with previously unrecognized malignancy, it was not incorporated into the final OncoScore because of incomplete data availability and limited evidence of incremental discrimination. Categorical BMI was available in 71.7% of patients; its addition increased the AUC from 0.7196 to 0.7372, without a significant improvement in discrimination (*p* = 0.2883). Continuous BMI was available in 57.9% of the derivation cohort and showed a larger numerical increase in AUC, from 0.7118 to 0.7486, although this difference also did not reach statistical significance (*p* = 0.0576). In addition, BMI was most often calculated from height and weight reported in the medical history rather than directly measured during hospitalization, which may have introduced additional measurement error. Thus, while BMI appears to provide additional predictive information, its substantial missingness, potential measurement error, and the absence of a robust improvement in discrimination did not support its incorporation into the present point-based score. Its potential contribution should be reassessed in future cohorts with more complete BMI data.

### 4.3. Performance Across Clinical Subgroups

In the exploratory subgroup analysis, the OncoScore showed numerically higher discrimination for malignancies not detectable on CTPA than for CTPA-detectable malignancies (AUC 0.723 vs. 0.681). This finding may be clinically plausible, as malignancy-related abnormalities already visible on CTPA may reduce the incremental value of laboratory-based risk stratification. Given the limited number of malignancies in each subgroup, this observation should be considered hypothesis-generating. However, this exploratory classification based on CTPA detectability does not provide a comprehensive description of the mode of detection of all newly diagnosed malignancies.

Although the OncoScore was derived exclusively in patients with newly diagnosed malignancy versus those without malignancy, exploratory evaluation in patients with previously known malignancy was considered justified because the two malignancy groups exhibited remarkably similar baseline characteristics. The only significant difference observed was a lower hemoglobin concentration among patients with established malignancy, likely reflecting the effects of advanced disease and anticancer treatment. Nevertheless, the discriminatory performance of the score was lower in patients with previously known malignancy (AUC 0.660) and this likely reflects the substantial heterogeneity of patients with established cancer. Unlike newly diagnosed malignancies, known cancers encompass a broad spectrum of disease stages, treatment statuses, therapeutic interventions, and tumor burdens, all of which may independently influence laboratory parameters. Excluding patients who had undergone recent surgery resulted in only a modest improvement in discrimination (AUC 0.672), suggesting that postoperative inflammatory changes alone do not explain the lower performance of the score in patients with established malignancy.

Taken together, these findings suggest that the OncoScore performs best in the clinical setting for which it was originally developed, namely the identification of previously unrecognized malignancy at the time of acute pulmonary embolism.

### 4.4. Comparison with Previous Prediction Models

The most extensively evaluated prediction model developed for occult cancer after VTE is the RIETE score, which incorporates several clinical variables, including age, sex, chronic lung disease, anemia, thrombocytosis, postoperative status, and previous VTE [15]. Although both models aim to facilitate cancer detection in patients with VTE, they differ in both predictor selection and intended clinical application. The RIETE score was developed to predict occult cancers diagnosed during follow-up after VTE, whereas the OncoScore was specifically designed to estimate the likelihood of malignancy being diagnosed during the index hospitalization for acute pulmonary embolism. Furthermore, the OncoScore relies exclusively on routinely available laboratory parameters, allowing immediate application at hospital admission without the need for detailed clinical history. More recently, Jara-Palomares et al. developed and externally validated the CAVE score, a prediction model for occult cancer diagnosed within one year after VTE. Similarly to the original RIETE score, the CAVE model was designed to predict cancer diagnosed during subsequent follow-up rather than malignancy diagnosed during the index hospitalization [32].

Subsequent external validation studies demonstrated more modest discriminatory performance of the RIETE score than originally reported, highlighting the ongoing need for improved risk stratification strategies in this clinical setting [19,33]. Rather than replacing the RIETE score, the OncoScore represents a complementary approach based on a different clinical objective and predictor domain. Whereas the RIETE score combines clinical characteristics and laboratory variables to estimate the risk of occult cancer diagnosed during follow-up after VTE, the OncoScore relies exclusively on routinely available laboratory parameters to estimate the likelihood of malignancy being diagnosed during the index hospitalization. Consequently, the two models should be regarded as complementary rather than competing approaches to cancer risk assessment after VTE.

### 4.5. Limitations

The present study has several limitations. The present analysis was retrospective and based on observational registry data and is therefore subject to the inherent limitations of this study design. Although the study included a large overall cohort, the number of patients with malignancy diagnosed during the index hospitalization was relatively limited. Given the low frequency of this outcome, the Brier score should be interpreted cautiously, as this measure is influenced by outcome prevalence.

Cancer diagnostic work-up was not fully standardized across centers or throughout the study period, which may have introduced heterogeneity in malignancy ascertainment. Moreover, patients with clinical or laboratory findings suggestive of malignancy may have undergone more extensive diagnostic evaluation, potentially increasing the likelihood of malignancy detection and introducing ascertainment bias. Detailed patient-level data on the specific mode of cancer detection were not systematically available. The REPER does not systematically capture incident cancer diagnoses during the year following PE. Consequently, some patients classified as having no malignancy during the index hospitalization may have been diagnosed with cancer during subsequent follow-up, introducing a potential risk of outcome misclassification. Overall, the OncoScore should be considered a preliminary derivation-stage model requiring independent external validation before clinical implementation.

In addition, provoked/unprovoked PE status in the registry was assigned retrospectively at discharge, with newly diagnosed malignancy itself considered a provoking factor; therefore, this variable could not be used for an unbiased subgroup analysis of OncoScore performance according to provoked versus unprovoked PE. Accordingly, the OncoScore was derived in an unselected acute PE population, and its performance has not been specifically evaluated in patients with unprovoked PE.

The initial reduction in candidate predictors based on univariable statistical significance may have excluded potentially informative variables and contributed to predictor-selection instability. Although bootstrap validation of the fixed final score showed minimal optimism, repetition of predictor selection and CHAID cut-point determination yielded a lower optimism-corrected AUC, indicating instability in the data-driven development process.

Finally, although an exploratory analysis was performed in patients with previously known malignancy, this subgroup was not representative of the intended target population for the score because disease stage and anticancer treatment may substantially influence the included laboratory parameters.

Despite these limitations, the study has several important strengths. It was based on a large multicenter registry of patients with objectively confirmed pulmonary embolism, enhancing the representativeness of the study population. Furthermore, the OncoScore relies exclusively on routinely available laboratory parameters, making it simple, inexpensive, and potentially easy to implement in routine clinical practice without additional testing.

## 5. Conclusions

The OncoScore represents a simple laboratory-based tool for estimating the probability of previously unrecognized malignancy in patients presenting with acute pulmonary embolism. In this derivation cohort, the score showed moderate discrimination and identified a subgroup with a low observed prevalence of newly diagnosed malignancy during the index hospitalization. Because the score relies exclusively on routinely available laboratory parameters, it is inexpensive and readily applicable at hospital admission, and it may facilitate risk stratification without additional testing. The OncoScore should be considered a preliminary derivation-stage model. A low score does not rule out occult malignancy and should not, by itself, justify withholding clinically indicated investigation or subsequent follow-up. Independent external validation is required before routine clinical implementation.

## Figures and Tables

**Table 1:** Baseline characteristics of patients according to malignancy status.

Characteristics	Newly Diagnosed Malignancy (n = 91)	Known Malignancy (n = 265)	No Malignancy (n = 2259)	Overall *p* *	Newly Diagnosed vs. Known *p* *
Age, mean ± SD	65.0 ± 13.1	66.7 ± 11.3	64.3 ± 16.1	0.061	0.239
Male sex, n (%)	40 (44.0%)	119 (44.9%)	1071 (47.4%)	0.620	0.875
**Comorbidities**, n (%)					
Chronic heart failure	13 (14.3%)	31 (11.7%)	355 (15.7%)	0.218	0.518
Arterial hypertension	50 (55.6%)	142 (53.8%)	1396 (61.9%)	0.021	0.771
Coronary artery disease	10 (11.1%)	32 (12.1%)	295 (13.1%)	0.788	0.798
Chronic lung disease	10 (11.1%)	34 (12.8%)	236 (10.5%)	0.492	0.669
Diabetes mellitus	12 (13.3%)	56 (21.1%)	462 (20.5%)	0.240	0.104
eGFR < 60 mL/min/1.73 m^2^	32 (35.2%)	96 (36.8%)	757 (34.3%)	0.716	0.782
eGFR < 30 mL/min/1.73 m^2^	8 (8.8%)	38 (14.4%)	177 (7.9%)	0.002	0.170
Anemia †	37 (40.7%)	132 (50.2%)	537 (24.0%)	<0.001	0.117
BMI category ‡				<0.001	0.760
– Normal weight (18.5–24.9 kg/m^2^)	43 (65.2%)	116 (61.1%)	680 (42.0%)	<0.001	
– Overweight (25.0–29.9 kg/m^2^)	15 (22.7%)	52 (27.4%)	590 (36.4%)	0.005	
– Obese (≥30.0 kg/m^2^)	8 (12.1%)	22 (11.6%)	350 (21.6%)	0.001	
**ESC risk category** **(4 strata)**				<0.001	0.168
– Low risk	16 (17.6%)	50 (18.9%)	699 (30.9%)		
– Intermediate–low	32 (35.2%)	68 (25.7%)	559 (24.7%)		
– Intermediate–high	31 (34.1%)	88 (33.2%)	690 (30.5%)		
– High risk	12 (13.2%)	59 (22.3%)	311 (13.8%)		
**Metastatic disease**	29 (31.9%)	82 (30.9%)		-	0.886
**Laboratory parameters**, median (Q1–Q3)					
D-dimer (mg/L)	5.52 (2.51–12.55)	4.91 (2.50–10.00)	4.33 (2.21–8.89)	0.048	0.452
CRP (mg/L)	68.10 (24.75–125.75)	62.70 (23.00–130.50)	39.60 (14.70–88.23)	<0.001	0.967
Hemoglobin (g/L)	125.00 (107.00–136.00)	116.50 (101.00–129.00)	133.00 (119.00–146.00)	<0.001	0.015
Leukocytes (×10^9^/L)	10.70 (8.20–15.90)	10.00 (7.40–14.00)	10.00 (7.80–12.80)	0.228	0.178
Platelets (×10^9^/L)	250.00 (199.00–303.00)	218.00 (163.00–322.50)	224.50 (179.00–282.00)	0.062	0.079

BMI, body mass index; CRP, C-reactive protein; ESC, European Society of Cardiology; eGFR, estimated glomerular filtration rate; SD, standard deviation; Q1–Q3, first and third quartiles. * Overall *p* represents comparison among all three study groups, whereas Newly diagnosed vs. Known *p* represents pairwise comparison between patients with newly diagnosed and previously known malignancy. † Anemia was defined according to the predefined REPER (Regional Pulmonary Embolism Registry) definition using sex-specific hemoglobin thresholds (<125 g/L in men and <115 g/L in women). Percentages were calculated using the number of patients with available data for the respective variable as the denominator. ‡ BMI data were available in 1876 patients (71.7% of the cohort).

**Table 2:** Univariate logistic regression analysis of candidate predictors for newly diagnosed malignancy.

Variable	OR	95% CI	*p*
Age (per 1-year)	1.003	0.989–1.016	0.686
Male sex	0.87	0.57–1.33	0.518
Arterial hypertension	0.768	0.503–1.174	0.223
Chronic heart failure	0.893	0.491–1.624	0.711
Coronary artery disease	0.829	0.425–1.618	0.583
Chronic lung disease	1.071	0.547–2.095	0.841
Diabetes mellitus	0.598	0.323–1.108	0.102
BMI †	—	—	Overall *p* = 0.004
eGFR < 60 mL/min/1.73 m^2^	1.040	0.671–1.614	0.860
eGFR < 30 mL/min/1.73 m^2^	1.121	0.534–2.354	0.762
Creatinine (per 1 µmol/L)	0.999	0.995–1.003	0.544
D-dimer (per 1 mg/L)	1.013	0.993–1.033	0.200
CRP (per 1 mg/L)	1.002	1.000–1.004	0.049
Leukocyte count (per 1 × 10^9^/L)	1.033	1.008–1.058	0.008
Platelet count (per 1 × 10^9^/L)	1.002	1.000–1.004	0.019
Hemoglobin (per 1 g/L)	0.977	0.968–0.987	<0.001

† BMI: Overweight vs. normal: OR 0.433 (95% CI 0.238–0.786), *p* = 0.006; obese vs. normal: OR 0.398 (95% CI 0.185–0.855), *p* = 0.018.

**Table 3:** Univariate logistic regression analysis of OncoScore components for prediction of newly diagnosed malignancy.

Variable	Category	OR	95% CI	*p*
Hemoglobin	>142 g/L	Reference	—	—
	>103–≤142 g/L	3.86	1.83–8.12	<0.001
	≤103 g/L	8.86	3.86–20.32	<0.001
CRP	≤56 mg/L	Reference	—	—
	>56 mg/L	2.15	1.40–3.32	<0.001
Platelet count	≤225 × 10^9^/L	Reference	—	—
	>225 × 10^9^/L	1.86	1.19–2.91	0.006
Leukocyte count	≤16 × 10^9^/L	Reference	—	—
	>16 × 10^9^/L	2.59	1.53–4.39	<0.001

**Table 4:** Multivariable logistic regression analysis of OncoScore components for prediction of newly diagnosed malignancy, adjusted for age and sex.

Variable	Category	Adjusted OR	95% CI	*p*
Hemoglobin	>142 g/L	Reference	—	—
	>103–≤142 g/L	3.82	1.78–8.18	<0.001
	≤103 g/L	7.41	3.11–17.64	<0.001
CRP	≤56 mg/L	Reference	—	—
	>56 mg/L	1.59	1.01–2.50	0.046
Platelets	≤225 × 10^9^/L	Reference	—	—
	>225 × 10^9^/L	1.59	1.00–2.51	0.049
Leukocytes	≤16 × 10^9^/L	Reference	—	—
	>16 × 10^9^/L	2.17	1.25–3.76	0.006
Age	per 1 year	1.00	0.99–1.01	0.929
Sex	female vs. male	0.79	0.50–1.23	0.293

**Table 5:** Prevalence of newly diagnosed malignancy across OncoScore risk categories.

Risk Category	Newly Diagnosed Malignancy n/N (%)	Mean Predicted Probability
Low risk (≤2)	11/987 (1.1%)	1.7%
Intermediate risk (3–4)	55/999 (5.5%)	4.7%
High risk (≥5)	22/203 (10.8%)	12.1%

**Table 6:** Diagnostic performance of the OncoScore for prediction of newly diagnosed malignancy using a cut-off value of ≥3 points.

Parameter	Value (%)	95% CI
Sensitivity	87.5	78.7–93.6
Specificity	46.5	44.3–48.6
PPV	6.4	5.1–7.9
NPV	98.9	98.0–99.4

NPV, negative predictive value; PPV, positive predictive value.

**Table 7:** Anatomical distribution and CTPA detectability of newly diagnosed malignancies during hospitalization for acute pulmonary embolism.

Anatomical Localization	Newly Diagnosed Malignancies, N = 91, n (%)	CTPA-Detectable, N = 45, n	Non-CTPA-Detectable, N = 46, n
Pulmonary	37 (40.7%)	37	0
Mediastinum	3 (3.3%)	3	0
Gastrointestinal	22 (24.2%)	1	21
– Colorectal	7 (7.7%)	1	6
– Stomach	3 (3.3%)	0	3
– Hepatic	5 (5.5%)	0	5
– Pancreatic	5 (5.5%)	0	5
– Other	2 (2.2%)	0	2
Hematological	4 (4.4%)	0	4
Urogenital	14 (15.4%)	1	13
– Renal/bladder	6 (6.6%)	0	6
– Prostate	2 (2.2%)	0	2
– Gynecological	6 (6.6%)	1	5
Breast	2 (2.2%)	0	2
Other	2 (2.2%)	0	2
Carcinoma of unknown primary	7 (7.7%)	3	4
Metastatic disease at diagnosis	29 (31.9%)		

Metastatic disease is reported as an additional characteristic and is not mutually exclusive with the anatomical localization categories.

## Data Availability

The data generated in the present study may be requested from the corresponding author.

## Supplementary Materials

The following supporting information can be downloaded at: https://www.mdpi.com/article/10.3390/cancers18213440/s1, Table S1: Missing data for candidate predictors in the OncoScore derivation cohort; Figure S1: Decision-curve analysis of the OncoScore; Figure S2: ROC curve of the OncoScore for prediction of previously known malignancy; Figure S3: ROC curve of the OncoScore for prediction of previously known malignancy after exclusion of patients who underwent surgery within six months prior to pulmonary embolism.
