# Supplementary material for: Development of the OncoScore: A Diagnostic Prediction Score for Previously Unrecognized Malignancy in Patients with Acute Pulmonary Embolism
Source: Cancers (Basel). 2026 Oct 24;18(21):3440. doi: 10.3390/cancers18213440 (PMC13761907; doi:10.3390/cancers18213440)
Supplement: Supplementary file 1 [file cancers-18-03440-s001.zip › cancers-4607318-supplementary.pdf]

Supplementary Table S1. Missing data for candidate predictors in the OncoScore derivation cohort.

Candidate predictor	Missing, n	Missing, %	Available, n
Age	0	0.0%	2350
Sex	0	0.0%	2350
Arterial hypertension	6	0.3%	2344
Chronic heart failure	2	0.1%	2348
Coronary artery disease	8	0.3%	2342
Chronic lung disease	2	0.1%	2348
Diabetes mellitus	2	0.1%	2348
BMI category	664	28.3%	1686
eGFR <60 mL/min/1.73 m^2	50	2.1%	2300
eGFR <30 mL/min/1.73 m^2	23	1.0%	2327
Creatinine	22	0.9%	2328
D-dimer	179	7.6%	2171
CRP	106	4.5%	2244
Leukocyte count	20	0.9%	2330
Platelet count	69	2.9%	2281
Hemoglobin	20	0.9%	2330

Decision-curve analysis was performed in the complete-case cohort of 2,189 patients. Net benefit is shown across a range of threshold probabilities for an OncoScore-guided strategy compared with strategies of investigating all patients or investigating none (Supplementary Figure S1).

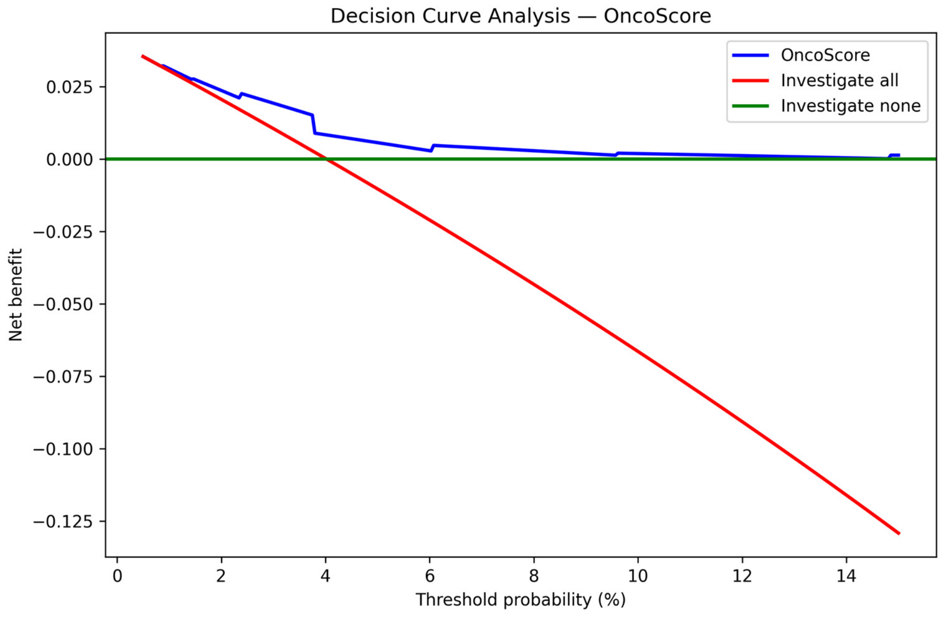

Supplementary Figure S1. Decision-curve analysis of the OncoScore.

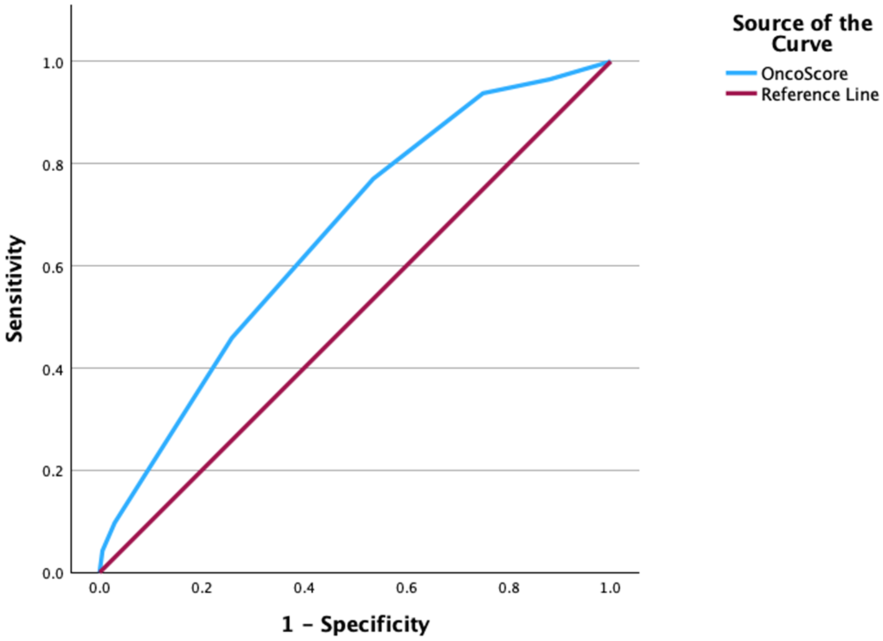

Supplementary Figure S2. ROC curve of the OncoScore for prediction of previously known malignancy.

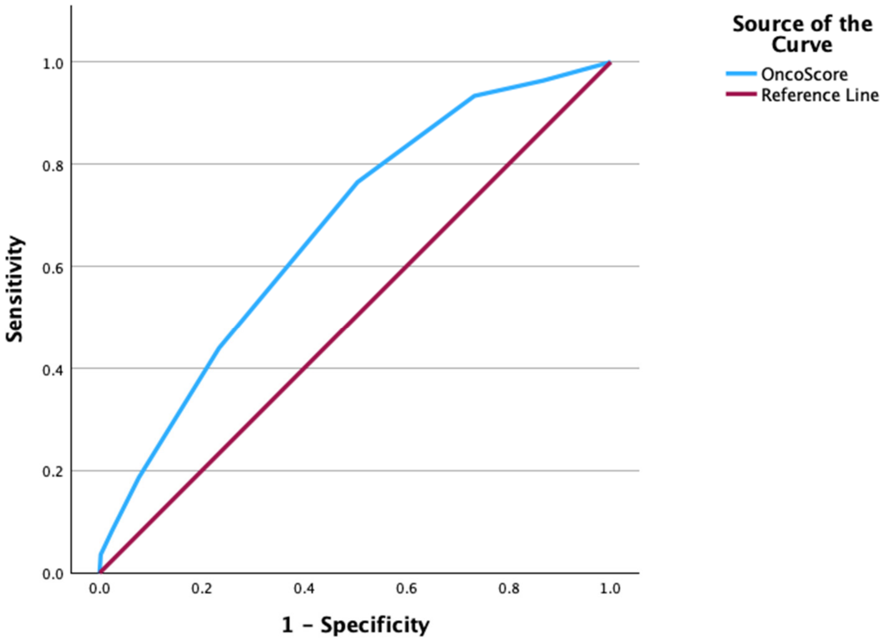

Supplementary Figure S3. ROC curve of the OncoScore for prediction of previously known malignancy after exclusion of patients who had undergone surgery within six months prior to pulmonary embolism.
